# Serious life events and associated PTSD in Gambian girls exposed to female genital cutting

**DOI:** 10.3389/fpubh.2023.1242270

**Published:** 2023-10-17

**Authors:** Trond Heir, Bothild Bendiksen, Fabakary Minteh, Rex A. Kuye, Inger-Lise Lien

**Affiliations:** ^1^Norwegian Centre for Violence and Traumatic Stress Studies, Oslo, Norway; ^2^Institute of Clinical Medicine, University of Oslo, Oslo, Norway; ^3^Department of Public & Environmental Health, University of the Gambia, Serekunda, Gambia

**Keywords:** female genital mutilation (cutting), PTSD – posttraumatic stress disorder, traumas and stressors, child health & development, epidemiol prevention, epidemiology – analytic (risk factors)

## Abstract

**Introduction:**

Poor mental health, such as post-traumatic stress disorder (PTSD), has been reported after female genital cutting (FGC). However, data documenting adverse consequences of FGC have insufficiently considered confounding factors, such as other traumatising events. Here, we examined the extent to which FGC versus other serious life events disturbed Gambian girls subjected to FGC. We additionally assessed the prevalence of PTSD and the extent to which it was attributed to FGC versus other serious life events.

**Methods:**

We conducted a cross-sectional study with a community-based sample of 12 years-old Gambian girls who had been subjected to FGC (*N* = 125). Using structured interviews, we assessed serious life events and probable PTSD related to the event that the girls cited as bothering them the most.

**Results:**

Most of the girls reported several serious life events in addition to FGC, such as witnessing violence, experiencing violence or assaults, death of a close relative, and being exposed to natural disasters or serious accidents, for an average 4.5 events per girl. Around one-sixth of the girls (16.8%) stated that FGC was the event that currently bothered them the most, whereas the majority (75.2%) emphasised other experiences. The girls who said they were most troubled by other events reported more impaired daily functioning than those most bothered by FGC. Overall, we found a prevalence of probable PTSD of 19.2%. Of 24 PTSD cases, one was attributed to the experience of FGC, and the remaining 23 were attributed to other events.

**Conclusion:**

Our findings indicate that FGC is less important than other serious life events in explaining high rates of PTSD in Gambian girls. Associations established in the field between FGC and adverse mental health must be interpreted with caution because girls who have undergone FGC may be severely exposed to other traumatising events.

## Highlights

– What is already known: Women who have undergone female genital cutting (FGC) are vulnerable to mental health problems. Studies of this association, however, have insufficiently assessed confounding factors such as other traumatic events in the interpretation and communication of causality.
– What this study adds: A high prevalence of post-traumatic stress disorder in Gambian girls who have undergone FGC appears to be due to other traumatising events that were particularly prevalent in this population.
– What do the new findings imply?: Caution is urged when interpreting and communicating associations between FGC and mental health, as girls who have undergone FGC may have experienced other traumatising events that warrant attention and preventive efforts. Future research must consider a wide variety of serious life events when examining mental health among girls or women who have undergone FGC.

## Introduction

Worldwide, approximately 44 million girls under age 15 years have undergone female genital cutting (FGC) (1, 2). Although many countries have passed laws banning FGC, it is still practiced by several African tribes. In the Republic of The Gambia (The Gambia), the site of the present study, the prevalence rate of FGC is around 75% (3, 4). The FGC procedure involves partial or total removal of external female genitalia or other injury to the female genitalia for cultural, religious, or other nontherapeutic reasons. The World Health Organization classification differentiates among four types of FGC (5). Type I (clitoridectomy), type II (excision), and type III (infibulation) account for approximately 66, 26, and 8% of cases in The Gambia, respectively (6).

The immediate medical complications of FGC include excessive bleeding, pain, swelling of the genital tissues, urinary retention, and problems with wound healing (7, 8). In the longer term, women who have undergone FGC are at greater risk for gynaecological, sexual, and obstetric complications (8).

In contrast to a large body of literature on the physical health effects of FGC, knowledge is limited regarding the mental health consequences. In a 2019 systematic review, Abdalla and Galea (9) identified 16 studies that examined mental health associated with FGC, six of which presented the association between FGC and mental health as the sole research question. Overall, the studies showed a higher burden of adverse mental health outcomes among women who were subjected to FGC compared with women who were not. Some studies have shown a correlation between the severity of FGC and the severity of adverse mental health outcomes. As the authors noted, the included studies had small sample sizes and did not consider confounding factors, such as traumatic life events such as being exposed to actual or threatened death, serious injury, violence or sexual assault. Thus, it cannot be ruled out that the adverse mental health outcomes are the result of other conditions that disadvantage women subjected to FGC.

Some studies have addressed various aspects of mental health among women and adolescent girls who have undergone FGC, including post-traumatic stress disorder (PTSD), depression, anxiety, somatisation, insomnia, helplessness, irritability, and low self-esteem (10–14). The premise of studies investigating PTSD, e.g., (11, 14–18) has been that the FGC procedure can represent a threat to the child’s physical integrity and be traumatic through inflicting pain and harm. None of these studies investigated whether the symptoms of PTSD could be related to other traumatic events in the lives of these girls and women, which is important because, for example, more severe PTSD could potentially relate to multiple traumatic experiences. To gain deeper insight into this issue, we investigated serious life events experienced by 12 years-old Gambian girls. Our aim was first to examine the extent to which FGC relative to other serious life events was reported as the event that currently bothered the girls the most. Our second aim was to examine the prevalence of PTSD in this population and the extent to which PTSD was attributed to FGC.

## Methods

### Participants

We used a subset of data from a cohort of 251 Gambian girls out of 300 girls aged 12 years who were invited to participate in a study of mental health among Gambian preteen girls, for an acceptance rate of 83.7%. The girls were recruited from 23 public schools in urban areas of The Gambia (19). A subsample of 134 girls had undergone FGC, and of this group, 125 (93.3%) who had completed a questionnaire about serious life events and whether they had post-traumatic stress symptoms, were included in the present study. All participants were Muslims affiliated with different ethnic groups, including Mandinka (*n* = 77), Fula (*n* = 22), Wolof (*n* = 11), Jola (*n* = 9), Serer (*n* = 2), Sarehule (*n* = 2), Jahanka (*n* = 1), and Bambara (*n* = 1). The recruitment of the overall cohort of 251 girls included a random selection procedure (19). Parents of selected girls received information about the study either at a meeting held at the school (about 40%), orally by phone (30%), or by written consent forms brought home by their daughters (30%). All parents signed consent forms, about 50% of them by fingerprint because of illiteracy.

### Measures

All study participants underwent a structured interview. Socio-demographic variables included age, number of siblings, and ethnic group. We did not capture information about parental education, occupation, income, or marital status because children in The Gambia often live in extended families within their households, and it can be difficult for a 12 years-old to single out one breadwinner. We interviewed the principal at each of the 23 schools about the background of participants at a group level. In general, girls in public schools (accounting for two-thirds of children in The Gambia) belong to families with low to moderate income and education.

#### Serious life events

For the girls who had underwent FGC, the structured interview assessed a history of the DSM-5 A criterion to identify potentially traumatic events experienced (20, 21). As a basis, we used a list of 15 categories of serious life events known to be potentially traumatising (i.e., natural disasters such as hurricanes or floods; fire; serious accidents; experiencing or witnessing violence at home or in the community, such as being slapped, punched, beaten, robbed by force, or attacked with a weapon; sexual abuse, defined as someone older touching the respondent’s private parts against her will or forcing her to have sex; scary medical procedures; war; or the violent or sudden unexpected death of a close relative) (21). Additionally, we included a category on FGC and the respondent’s age at the time of FGC. Respondents were asked if they had experienced each of the events directly, witnessed each of them, or had learned that the event happened to somebody close. The respondents were then asked to identify the event that currently bothered them the most.

#### Posttraumatic stress reactions

To assess posttraumatic stress symptoms, we used the Child PTSD Symptom Scale (CPSS) (22, 23). The CPSS indexes the frequency of 17 symptoms on a 4-point scale: (0) = not at all, (1) = once a week or less, (2) = 2–4 times a week, and (3) = 5 or more times a week. The scale includes five re-experiencing symptoms, seven avoidance symptoms, and five arousal symptoms. CPSS was designed for use in children aged 8–18 years (22, 23) and has been validated in samples of children aged 6–17 years (23). The scale has good psychometric properties, including internal consistency, test–retest reliability, and discriminant validity (22–24). In our sample, Cronbach’s Alpha for the CPSS scale was 0.79.

#### Daily functioning

In addition to questions about post-traumatic stress symptoms, the CPPS contains seven items that inquire about daily functioning (e.g., relationships with friends, family, schoolwork). The items were scored dichotomously as functional impairment being absent (0) or present (1) and yielded a total severity-of-impairment score ranging from 0 to 7.

#### PTSD prevalence

We scored the 17 symptom items of CPSS dichotomously to generate a DSM-IV-consistent diagnosis of PTSD (22). Participants who scored 2 or above on at least one of five re-experiencing symptoms, three of seven avoidance symptoms, and two of five arousal symptoms were classified as fulfilling the PTSD symptom criteria (22, 25). For a probable PTSD diagnosis, we also required functional impairment, defined as three or more of the seven functional impairment items being present (22). Cases with a positive A criterion and positive scores on the critical number of symptoms and functional impairment items in the CPPS were considered to have a DSM-IV-consistent diagnosis of PTSD.

#### Current physical complications

We asked whether the participants were bothered by genital pain, spent a long time urinating, or had pain when urinating (no or yes). Respondents giving a positive answer to at least one of these questions were characterised as having current physical complications.

### Procedure

An experienced researcher and child psychiatrist was responsible for a 3 days training of six research supervisors and 12 research assistants on the structured interview, including the questionnaire and checklists. The training took place at the Department of Public and Environmental Health, University of The Gambia. The research assistants who performed the data collection were all women at the University of The Gambia. All interviews were conducted in English; however, inquiries and ambiguities were explained in local languages. All interviews were conducted at an office or a classroom at the school, undisturbed and away from other students and teachers.

Data was punched directly into an SPSS file and stored in de-identified form, that is, research data and identifying elements were stored separately, and in such a way that the researchers only had access to research data. PCs with data files were not connected to the internet and they were stored in a secure location with access control when not in use. Only the researchers had access to the data files.

### Ethics statement

The study was approved by the Norwegian Regional Ethics Committee and The Gambia Government/MRC Joint Ethics Committee. Fingerprinted consent forms from illiterate parents following comprehensive oral information were considered informed consent. To deal with potential serious reactions among study participants, we used professional and trained interviewers and contact persons with mental health competence and ensured that the interviews were carried out in safe settings. A signed collaboration agreement was made with Faji Kunda Health Center in case of serious reactions to the interview.

Plenty of time was set aside after each interview for a conversation about health conditions. Trained interviewers were required to inform the participants about existing healthcare services and how to go about getting help. A contact person with healthcare expertise was always available for help and support.

### Analyses

We compared background characteristics, explanatory variables, and outcomes between girls who reported FGC as the event that bothered them the most and those who did not, using chi square and independent sample *t*-tests. All tests were two tailed, and differences were considered significant if *p* < 0.05. Data were analysed using SPSS version 25 for Windows (SPSS Inc., Chicago, IL, United States).

## Results

Overall, the Mandinka were the largest ethnic group, comprising more than half of the 125 participants (Table 1). Almost half of all participants (46.4%) had undergone FGC before age 4 years. At the age of 12 years, the participants had experienced a wide range of serious life events in addition to FGC (Table 2), with an average of 4.5 serious life events per individual. The events experienced by most participants, apart from FGC, were witnessing violence, experiencing violence or assault themselves, death of a close relative, and being exposed to a natural disaster or serious accident (Table 2).

**Table 1:** Background characteristics of 12 years-old Gambian girls who have undergone female genital cutting (FGC) in the total sample and in subsamples characterised by whether circumcision was the event that bothered the participants the most or not.

	Total sample (*N* = 125)	FGC bothers them the most (*n* = 21)	FGC did not bother them the most (*n* = 104)
	Mean (SD)	Mean (SD)	Mean (SD)
Age (year, month)	12.0 (0.48)	12.0 (0.3)	12.1 (0.5)
Number of siblings	4.4 (2.6)	5.2 (2.1)	4.3 (2.7)
PTSD symptom score	11.4 (6.7)	9.3 (5.7)	11.8 (6.8)
Daily functioning impairment score	1.4 (1.6)	0.67 (1.2)	1.5 (1.7)*

	*n* (%)	*n* (%)	*n* (%)
Ethnic group affiliation:			
Mandinka	77 (61.6)	16 (76.2)	61 (58.7)
Other^a^	48 (38.4)	5 (23.8)	43 (41.3)
Age at FGC ≥4 years	58 (46.4)	18 (85.7)	40 (38.5)**
Current physical complications^b^	37 (29.6)	6 (28.6)	31 (29.8)
PTSD prevalence	24 (19.2)	1 (4.8)	23 (22.1)

*p < 0.05 and **p < 0.001.

a Fula (*n* = 22), Wolof (11), Jola (9), Serer (2), Sarehule (2), Jahanka (1), Bambara (1).

b Current physical complications: genital pain, spending a long-time urinating, or having pain when urinating.

**Table 2:** Prevalence of serious life events, the event that bothered the participant the most, and events causing posttraumatic stress disorder (PTSD) in girls (*N* = 125) who had been exposed to female genital cutting (FGC).

	Serious life event experienced	Event that currently bothered the most	Event causing PTSD
	*n* (%)	*n* (%)	*n* (%)
FGC	125 (100)	21 (16.8)	1 (0.8)
Natural disaster or serious accident	65 (52.0)	6 (4.8)	2 (1.6)
Physical violence/assault	103 (82.4)	17 (13.6)	2 (1.6)
Witnessing violence	109 (87.2)	10 (8.0)	3 (2.4)
Unwanted sexual experience/abuse	11 (8.8)	2 (1.6)	0
Scary medical procedure	46 (36.8)	5 (4.0)	0
Death of a close relative	66 (52.8)	35 (28.0)	11 (8.8)
Another event^a^	35 (28.0)	19 (15.2)	5 (4.0)
Total	560	115 (92.0)	24 (19.2)

a Another event included having a brother who disappeared 6 months before (*n* = 1), bullying by children or teacher in class (*n* = 3), being kidnapped (*n* = 1), domestic fighting between parents (*n* = 2), physical illness (*n* = 2), separation from mother or father due to divorce of the parents (*n* = 4), or not specified (*n* = 22).

There was great variation in which type of event bothered the participants the most (Table 2). Around one-sixth of the girls (16.8%) stated that FGC was the event that currently bothered them the most, whereas the rest emphasised other experiences (75.2%) or did not report events that bothered them (8.0%). For example, 28.0% stated that the death of a close relative was the event that currently bothered them the most (Table 2).

Girls who cited FGC as the event that bothered them most differed significantly in some ways from the other participants (Table 1), including having undergone FGC at an older age, having better daily functioning, and possibly having a lower prevalence of PTSD (4.8% versus 22.1%, *p* = 0.065). These two groups did not differ in current physical complications, including genital pain, spending a long-time urinating, or having pain when urinating.

Overall, the prevalence of probable PTSD was 19.2%. Of 24 cases of PTSD, one (4.2%) was attributed to the experience of FGC, and the remaining 23 (95.8%) were attributed to other serious life events (Table 2).

## Discussion

Gambian girls who had undergone FGC had generally experienced a wide range of additional potential traumatic events that, to a greater extent than FGC, they rated as the event that currently bothered them the most. One in every six of the girls considered FGC as the serious life event currently bothering them the most. Although the prevalence of probable PTSD was high (19.2%), of 24 cases identified, one was linked to FGC and the other 23 attributed to stress reactions to other serious events.

The findings demonstrate the importance of considering a wide range of potential traumatic events when examining mental health in girls who have undergone FGC, as they may have experienced other traumatising events that warrant attention and preventive efforts. High rates of mental health problems among females who have undergone FGC, which has been documented in several studies (9), must be interpreted with caution because girls who have undergone FGC also may have been severely exposed to other traumatic events.

The overall prevalence of PTSD in our study was far higher than reported in general populations of children and adolescents in the United States and Europe (26–28). These differences may be linked to the high incidences of potentially traumatic events identified in the current study, such as the death of close relatives, exposure to violence, and experiences of natural disasters or serious accidents. The prevalence of serious life events among these girls was far higher than in population studies from the United States and Europe (26–28). FGC cannot be ruled out as contributing significantly to adverse mental health, but our findings indicate that other traumatic events or their accumulation may predominate as explanatory of high rates of PTSD among Gambian girls. Girls who have undergone FGC may also have experienced major challenges in terms of economic, social, or cultural burdens that can significantly affect mental health.

The link between age at FGC and FGC as the event that bothered girls the most may be related to changes in memory encoding, retention, and retrieval that occur during early childhood. Most people have no recollection of infancy and earliest childhood, but quantitative changes in basic memory processes can explain the fading of childhood amnesia during the third and fourth years of life (29). Although the timing of FGC may affect how the event is remembered later, the exceptionally low prevalence of PTSD related to FGC in the current work does not provide evidence in support of the hypothesis linking FGC timing and development of PTSD (18).

Girls who rated FGC as the event that bothered them the most had overall better daily functioning and a trend to a lower prevalence of PTSD compared with girls who rated other events as bothering them the most. This indicates that other events may have been a greater burden on functioning and mental health than FGC.

The finding that FGC was not the predominate trauma related to PTSD for most of these girls deserves some reflections regarding possible protective mechanisms. Psychological reactions in response to trauma depend on how an individual interprets the event, including being able to understand it in a meaningful context (30, 31). In the traditional circumcision of Gambian girls, the procedure is deeply rooted in a cultural context of ritual acts and celebration (32). The girl is being prepared to be the centre of a large family celebration where she will receive honour, gifts, and gratitude.

After the procedure, the girl undergoes formal instruction about the importance of the FGC and why it was carried out. She is repeatedly told that the procedure will turn her into a proud and honourable woman, so that she can get married, have children, and live a good and respectful life (32). The use of words and phrases such as “clean” and “being like your mother,” as well as tautological explanations such as “it’s necessary” and “that’s how we do it,” makes the learning more explicit. In line with knowledge that meaning-making processes are central to recovery and resilience (33), growing up with an integrated identity as a “cut, proud, and honourable” woman might contribute to the maintenance of mental health.

Second, traditional rituals following FGC may be effective in reducing pain and emotional stress. In their discussion of cultural protection against traumatic stress after FGC in Gambian girls, Schultz and Lien (32) argue that traditional rituals are rich in elements similar to the principles of modern empirically based crisis intervention. In keeping with five essential principles of trauma intervention described by Hobfoll and colleagues (34), the girls are provided with a sense of safety in the aftermath of the operation, and they are calmed down and encouraged through the healing period (32). Self-efficacy is empowered, as is the connectedness and feeling of belonging to the tradition, family, and female identity. A collective hope is linked to their new status, their position, and future identity (32).

Third, an ecological view of trauma recovery (35, 36) should be included. According to this view, the extensive social support provided in the procedure’s aftermath and the need to adapt to the cultural collectivist mindset may serve adaptive purposes and support mental health maintenance.

### Strengths and limitations

The strengths of the study include a community-based design, a study sample from a native population in which FGC was highly prevalent, and a high participation rate. The study is one of the few to have examined the associations between FGC and PTSD in young adolescent girls living in their country of origin. It was designed to assess a wide range of life events as a cause of PTSD, highlighting the event that bothered the participant the most. Trauma exposure and prevalence of PTSD were clinically assessed by a structured interview conducted by trained women who were students native to the region with competence in culture and language.

Our study has some important limitations. Retrospective reports of trauma exposure may introduce recall bias because potential traumatic events can be forgotten or reinforced in memory (37, 38). Cultural expectations not to be critical of FGC may have led some girls to feel reluctance about ranking FGC as the event that bothered them the most. Nevertheless, the low prevalence of PTSD among those most distressed by FGC and poorer daily functioning among girls who cited other trauma strengthens the conclusion that traumas other than FGC had a greater impact on mental health in most of these Gambian girls.

The study was conducted among 12 years-old girls in The Gambia, and the results cannot be easily generalised to other cultures or to circumcised women living away from their places of origin. Schultz and Lien (32) specify how cultural protection against traumatic stress after FGC is deeply rooted in the traditional cultural belief system. When a circumcised woman is exposed to another belief system that condemns or even criminalises the practice, her culturally encoded protections are strongly challenged. Circumcised women who live far from the supportive cultural context in their homeland may therefore be more vulnerable to adverse mental health related to FGC (10, 39).

We used a face-to-face interview based on the CPSS questionnaire to diagnose PTSD, in which wording of the DSM-IV criteria was adapted for children and adolescents, rather than using a structured clinical interview that is considered the gold standard for diagnosing mental disorders. Although a questionnaire based on DSM-IV has been shown to be equivalent to a structured clinical interview of DSM-IV in epidemiological research (40), the procedure carries the limitation of unknown direction of an eventual bias.

Finally, non-participation may have caused biased prevalence estimates. However, the high participation rate in the present study indicates that non-respondents would have to have had exceptionally deviant results to have significantly influenced the estimates given.

## Data availability statement

The datasets presented in this article are not readily available because Data are from the project “Physical and psychological healthcare for girls and women with Female Genital Mutilation/Cutting (FGM/C).” According to the approval from the Norwegian Regional Ethics Committee and the Gambia Government/MRC Joint Ethics Committee, the data are to be stored properly and in line with Norwegian and Gambian laws of privacy protection. Public availability would compromise privacy of the respondents. However, anonymised data are freely available to interested researchers upon request, pending ethical approval from our ethics committees: ethics@mrc.gm/post@helseforskning.etikk.no. Requests to access the datasets should be directed to TH (trond.heir@medisin.uio.no).

## Ethics statement

The studies involving humans were approved by The Norwegian Regional Ethics Committee, & The Gambia Government/MRC Joint Ethics Committee. The studies were conducted in accordance with the local legislation and institutional requirements. Written informed consent for participation in this study was provided by the participants’ legal guardians/next of kin.

## Author contributions

TH: analyzing data, data interpretation, and writing the draft. BB: planning study design, analyzing data, data interpretation, and writing the draft. FM and RK: planning study design, data interpretation, and actively contribute to the writing process. I-LL: organization of the research project, planning study design, data interpretation, and actively contribute to the writing process. All authors contributed to the article and approved the submitted version.
